# Immunohistochemical analysis and scRNA-Seq identifies vascular endothelial CXCR4 expression as a predictor of poor prognosis in pancreatic ductal adenocarcinoma

**DOI:** 10.1016/j.tranon.2026.102916

**Published:** 2026-07-14

**Authors:** Shunsuke Imamura, Takeshi Uehara, Mai Iwaya, Shiho Asaka, Shinsuke Sugenoya, Hiroshi Sawaguchi, Yasuhiro Kuraishi, Tomoyuki Nakajima, Shotaro Komamura, Akira Nakamura, Takefumi Kimura, Yugo Iwaya, Akira Shimizu, Yuji Soejima, Hiroyoshi Ota, Tadanobu Nagaya

**Affiliations:** a Division of Gastroenterology and Hepatology, Department of Medicine, Shinshu University School of Medicine, Matsumoto, Japan; b Department of Laboratory Medicine, Shinshu University School of Medicine, 3-1-1 Asahi, Matsumoto, Nagano, 390-8621, Japan; c Department of Laboratory Medicine and Pathology, Life Science Research Center, Nagano Children’s Hospital, Azumino, Japan; d Division of Gastroenterological, Hepato-Biliary-Pancreatic, Transplantation and Pediatric Surgery, Department of Surgery, Shinshu University School of Medicine, Matsumoto, Japan; e Department of Clinical Laboratory Sciences, Shinshu University School of Medicine, Matsumoto, Japan

**Keywords:** CXCR4, Invasive front, Pancreatic ductal adenocarcinoma, Vascular endothelium

## Abstract

**Background:**

The clinical significance of C-X-C chemokine receptor 4 (CXCR4) expression at the invasive front of pancreatic ductal adenocarcinoma (PDAC) remains unclear. This study investigated CXCR4 expression in the vascular endothelium at sites of cancer tissue infiltration into the pancreatic surrounding fatty tissue in resected PDAC specimens.

**Methods:**

We prepared tissue microarrays from 80 cases of PDAC resected between 2011 and 2023 and retrospectively analyzed the samples by immunohistochemistry. CXCR4 expression in blood vessels was semi-quantitatively scored (0 to 3+) and categorized as high (2–3+) or low (0–1+). We also analyzed the clinicopathological features associated with CXCR4 expression. Finally, leveraging public databases for single-cell RNA sequencing was used to confirm the localization of CXCR4 expression.

**Results:**

Of the 80 cases, 33 exhibited high CXCR4 expression and 47 showed low expression. The high expression group had a significantly shorter median overall survival (469 versus 1176 days, P = 0.0361). In the multivariate analysis, high CXCR4 expression (hazard ratio 1.8180, 95% confidence interval, 1.0350–3.193, P = 0.0342) was an independent prognostic factor. single-cell RNA sequencing revealed sparse CXCR4 expression in arteries, veins, capillaries, and tip-like endothelial cells.

**Conclusion:**

Vascular endothelial CXCR4 expression at the invasive front of PDAC is associated with poor prognosis and may be associated with tumor progression in correlation with angiogenic mechanisms.

## Highlights

• Vascular endothelial CXCR4 expression is a novel poor prognostic factor for PDAC.
• Immunohistochemistry and scRNA-seq confirmed CXCR4 expression in endothelial cells.
• High endothelial CXCR4 expression correlates with shorter PDAC patient survival.

## Introduction

Pancreatic cancer is one of the most lethal malignancies. Most pancreatic cancers are ductal adenocarcinomas (PDAC) [1]. Adjuvant and neoadjuvant chemotherapies as well as surgical resection are common for stage II or higher [2]; however, 5-year survival remains in the low teens [3]. Thus, identifying prognostic factors in surgical specimens is a critical issue, and evaluating the expression of various factors within PDAC tissues and the surrounding stroma may provide insight into potential therapeutic targets.

C-X-C chemokine receptor type 4 (CXCR4), through interaction with its ligand stromal cell–derived factor 1 (SDF-1)/CXC motif chemokine 12 (CXCL12), is involved in various biological processes related to cancer progression, such as cell migration, infiltration, survival, and angiogenesis. Previous studies have established that CXCR4 is frequently overexpressed in PDAC, which correlates with poor survival and increased metastatic potential [4,5]. Specifically, a meta-analysis by Krieg et al. confirmed that CXCR4 expression serves as a robust prognostic biomarker in PDAC [6]. Furthermore, CXCR4 has also been reported to induce the production of angiogenic factors from vascular endothelial cells [7,8], and its signaling pathway plays a critical role in the recruitment of immunosuppressive cells, contributing to an "immune-cold" tumor microenvironment [9,10].

While the role of CXCR4 in tumor cells is well-documented, its expression in the vascular endothelium—particularly at the invasive front—remains less explored in PDAC. Given that CXCR4 inhibition has shown promise in clinical settings, such as the COMBAT trial [11] where it was combined with pembrolizumab to enhance anti-tumor immunity, understanding the precise localization of CXCR4 is increasingly vital. To date, no study has examined CXCR4 expression in the vascular endothelium at the site of tumor infiltration in PDAC, nor has there been any report examining its association with clinical findings. It is important to clarify how CXCR4 expression in the tumor vascular endothelium affects PDAC progression and prognosis to elucidate the pathogenesis of PDAC and aid the search for new therapeutic targets.

This study provides a spatially focused evaluation of CXCR4 expression specifically in the vascular endothelium at the PDAC invasive front and assesses its clinical significance through immunohistochemistry (IHC) and single-cell analysis.

## Materials and methods

### Patients

Among PDAC cases resected at Shinshu University from 2011 to 2023, 80 cases with good specimen condition, positive control, and predominant invasive cancer were included in the analysis. A total of 82 cases were initially considered; however, 1 case each was excluded due to non-invasive cancer or poor staining. The following clinicopathological data were obtained: patient gender and age, localization, histological grade, tumor size, lymphatic invasion, venous invasion, perineural invasion, epithelial–mesenchymal transition (EMT) phenotype, tumor-infiltrating lymphocyte (TIL) grade, lymph node metastasis, tumor markers, cancer stage, chemotherapy, and patient outcome. Tumor stage and histology were reassessed using the staging systems of the eighth edition of the Union for International Cancer Control Tumor, Node, Metastasis (TNM) classification system [12] and the fourth edition of the World Health Organization Classification of Tumors [13]. Overall survival (OS) was defined as the duration from the date of surgical resection to either death or the last follow-up. This study adhered to the ethical principles outlined in the Declaration of Helsinki and received approval from the Clinical Trial Review Committee of Shinshu University School of Medicine (approval number: 5836).

### Histopathology and IHC

We used surgically resected, formalin-fixed paraffin-embedded PDAC specimens. Optimal lesions where cancer tissue infiltration into the pancreatic surrounding fatty tissue were selected after assessing hematoxylin and eosin-stained sections. Tissue microarrays (TMAs) were created by punching out tissue cores (3 mm) from each donor tumor block using thin-walled stainless steel needles (Azumaya Medical Instruments, Tokyo, Japan) and assembling them into recipient paraffin blocks [14,15]. Serial sections (4 μm) were cut from the TMA blocks and stained with hematoxylin and eosin. Two pathologists (T.U. and M.I.) re-evaluated the histopathological findings. TIL grade was assessed at the site of PDAC infiltration into the pancreatic surrounding fatty tissue and classified as follows: 0, <10 lymphocytes observed per high-power field of view (HPF); 1, 10–50 lymphocytes per HPF; 2, 51–100 lymphocytes per HPF; and 3, >100 lymphocytes per HPF [9,14].

Immunostaining for E-cadherin (NCH-38; prediluted; Dako, Carpinteria, CA, USA) or vimentin (V9; prediluted; Dako, Carpinteria, CA, USA) was performed using the Dako Omnis automated staining platform (Agilent Technologies, Carpinteria, CA, USA).

E-cadherin expression was scored based on the percentage of positive cells as follows: 0 (<10% of tumor cells or no staining), 1 (10%–49% positive), 2 (50%–70% positive), and 3 (>70% positive). Samples with scores of 0 and 1 were categorized as E-cadherin–negative, and those with scores of 2 and 3 were categorized as E-cadherin–positive​ [16]. For vimentin, clear cytoplasmic staining was considered positive. EMT phenotypes were categorized into three groups as described by Aruga et al. [17]: non-EMT (E-cadherin–positive, vimentin-negative), incomplete EMT (E-cadherin–negative and vimentin-negative or E-cadherin–positive and vimentin-positive), and complete EMT (E-cadherin–negative and vimentin-positive). The incomplete EMT and complete EMT groups were analyzed together as the EMT phenotype, and the non-EMT group was analyzed separately.

To characterize the localization of CXCR4 within the tumor microenvironment, serial sections were also stained for CD34 (clone QBEnd 10; prediluted; Dako) as a vascular endothelial marker and D2–40 (clone D2–40; prediluted; Dako) as a lymphatic endothelial marker. These stains were performed on the Dako Omnis platform using the manufacturer’s recommended protocols. The use of these markers allowed for the definitive identification of vascular versus lymphatic endothelial cells in the areas where CXCR4 expression was evaluated.

### IHC for CXCR4

Immunohistochemical staining for CXCR4 was conducted using a primary antibody (clone UMB2; dilution 1:2000; Abcam, Cambridge, UK). For antigen retrieval, we heated the samples in EDTA buffer (pH 9.0) in the microwave for 20 min. Immunostaining was automated using a Dako Omnis platform. The staining intensity of CXCR4 in tumor and stromal cells was evaluated manually using a semi-quantitative four-tier scale based on the morphological criteria described by Price et al. [18]: 0 (no staining; no discernible 3,3′-diaminobenzidine chromogen), 1+ (weak; faint brown staining visible only at high magnification), 2+ (moderate; distinct brown staining where the blue hematoxylin nuclear counterstain remains clearly visible), and 3+ (intense; strong dark brown staining that obscures or nearly obscures the nuclear counterstain). To ensure the objectivity of the four-tier scoring system, representative images of each intensity score (0, 1+, 2+, and 3+) were defined as shown in Fig. S1. For analytical purposes, the scores were categorized into two groups: low CXCR4 expression (scores 0 and 1+) and high CXCR4 expression (scores 2+ and 3+).

### Single-cell RNA sequencing analysis of CXCR4 expression in PDAC samples

Single-cell RNA sequencing (scRNA-seq) analysis of CXCR4 expression was conducted using a publicly available dataset (accession number: GSE205013) in the NCBI Gene Expression Omnibus (GEO) database, as previously described by Werba et al. [19] and Benitz et al. [20]. This dataset comprised primary and metastatic (liver) human PDAC tumors, some pre-treatment and some post-chemotherapy. Data processing and analysis were conducted using the Seurat R package (v4.1.0 or v5.0). Raw count matrices were normalized to a total expression of 10,000 molecules per cell and subsequently scaled. We identified highly variable genes across cells and applied principal component analysis to reduce dimensionality, selecting the top 30 principal components based on the JackStraw and Elbow plots. To assess cellular similarities, the FindNeighbors function (using the 30 PCs) and the FindClusters function (with a resolution of 0.8) were employed. We visualized cellular heterogeneity by applying uniform manifold approximation and projection (UMAP) using the runUMAP function with parameters set to n.neighbors = 30 and min.dist = 0.3. To ensure the reliability of endothelial cell identification, cells expressing canonical endothelial markers, such as PECAM1 and VWF, were first identified and sub-clustered. Overall, 4302 endothelial cells integrated across 17 distinct batches from the GSE205013 dataset were employed for the subsequent analysis, including the characterization of endothelial sub-populations shown in Fig. 3. A detailed summary of the dataset composition containing the number of cells per batch and sample types is provided in Table S1. The expression distribution of multiple endothelial marker genes was assessed across single-cell clusters derived from PDAC tissue to classify vascular endothelial subtypes. Using previously reported criteria [7], we annotated endothelial cells as follows: arterial endothelial cells (expressing GJA5, FBLN5, and LTBP4), venous endothelial cells (expressing ACKR1, SELP, and VCAM1), capillary endothelial cells 1 (expressing CA4, FCN3, and EDN1), capillary endothelial cells 2 (expressing CA4 and EMCN), tip-like endothelial cells (expressing CXCR4, ESM1, and ANGPT2), and lymphatic endothelial cells (expressing PROX1).

To identify the molecular signature of CXCR4-expressing vasculature, the 4302 endothelial cells were categorized into CXCR4-positive (n = 878) and CXCR4-negative (n = 3424) groups based on CXCR4 transcript levels. Differential gene expression (DGE) comparisons between the groups was performed using the Wilcoxon rank-sum test via the FindMarkers function. Statistical significance was assessed using both nominal P-values and adjusted P-values (Bonferroni correction) to characterize the transcriptomic shifts. To evaluate the biological plausibility of our clinical findings, the expression levels and frequencies of pro-angiogenic markers, including VEGFA and PECAM1, were compared between the groups as well.

### Statistical analysis

Fisher’s exact test or the Wilcoxon rank-sum test was used to evaluate differences between patient subgroups. Survival was estimated using the Kaplan–Meier method and compared using the log-rank test. Differences for which the P-value was <0.05 were considered significant. Univariate and multivariate Cox proportional hazards regression were performed to determine predictors of survival. To ensure the validity of the Cox models, the proportional hazards assumption was verified using Schoenfeld residuals. Statistical analyses were conducted using R software version 4.1.3​ (The R Foundation, Vienna, Austria). In multivariate analysis, the number of items was set according to the sample size (n = 80) to prevent overfitting. Variables with a P-value <0.1 in univariate analysis that were also considered clinically significant were included, while carefully avoiding multicollinearity as assessed by variance inflation factors. Inter-observer agreement for IHC scoring was assessed using Cohen’s kappa statistics.

### Data availability

The scRNA-seq data used in this study are publicly accessible via the NCBI GEO under accession number GSE205013, and can be retrieved at https://www.ncbi.nlm.nih.gov/geo/query/acc.cgi?acc=GSE205013. Data processing and analysis were performed using the Seurat package (v4.1.0) [21].

## Results

### Expression of CXCR4 in PDAC samples

CXCR4 expression was detected in multiple cell types at sites of cancer tissue infiltration into the pancreatic surrounding fatty tissue (Fig. S2) in resected PDAC specimens (Fig. S3A, B) but was mainly observed in the vascular endothelium (Fig. S3B), as confirmed by the co-expression of CD34 (Fig. S3C) and absence of D2–40 expression (Fig. S3D). Furthermore, CXCR4 expression was mostly low or weak in tumor cells in the invasive front, whereas it was more pronounced in the vasculature. Scores of 0/1/2/3 were observed in the tumor cells of 32/42/2/4 cases, respectively, and in the vascular endothelial cells of 30/17/23/10 cases, respectively (P = 0.00000525) (Table 1). On the basis of this observation, and previous reports [7] that CXCR4 induces the production of angiogenic factors from vascular endothelial cells, we selected vascular endothelial cells for further analysis to assess the frequency of CXCR4 positivity in areas containing vessels. In all 80 cases analyzed, CXCR4 expression was identified within the vasculature of the tumor stroma; the cases were classified into high and low CXCR4 expression groups.

**Table 1:** CXCR4 expression in vascular cells at the infiltrating front of pancreatic ductal adenocarcinoma.

**Staining score**	**Number of cases by localization of CXCR4 expression**		**P-value**
	Vascular endothelial cells	Tumor cells	
			**0.00000525**
0, no staining	30	32	
1, weak staining	17	42	
2, moderate staining	23	2	
3, intense staining	10	4	

### Correlation between CXCR4 expression and clinicopathological features

**Fig. 1:**
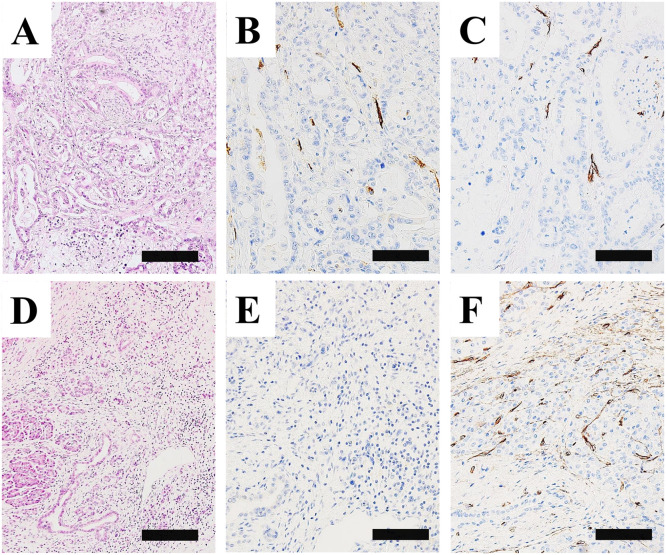
CXCR4 expression and CD34 expression. Distinct areas exhibiting high CXCR4 expression (A) and low CXCR4 expression (D). Section regions were classified by CD34 expression (C: high expression, F: low expression); the corresponding expression of CXCR4 in those regions is presented in B (high expression, corresponding to C) and E (low expression, corresponding to F). A and D, hematoxylin-eosin staining; B and E, CXCR4 immunostaining; and C and F, CD34 immunostaining. Bar indicates 200 μm in panels A and D, and 100 μm in panels B, C, E, and F.

The clinicopathological characteristics of the 80 patients with PDAC are summarized in Table 2. Immunohistochemical analysis revealed that 33 (41%) of the 80 PDAC cases exhibited high CXCR4 expression, while 47 cases (59%) showed low expression. Cohen’s kappa for the inter-observer agreement of CXCR4 scoring was high at 0.87 (95% confidence interval, 0.76–0.98). Representative expression patterns are shown in Fig. 1A (high expression) and Fig. 1D (low expression). Immunohistochemical staining images of CXCR4 expression are shown in Fig. 1B (high expression) and E (low expression). Interestingly, CXCR4 expression was observed in areas adjacent to CD34-positive vascular endothelial cells (Fig. 1C: high expression, F: low expression).

**Table 2:** CXCR4 expression and clinicopathological characteristics.

Factors		Total	CXCR4		P-value
			Low	High	
		(n = 80)	(n = 47)	(n = 33)	
Age (years)	Median (Range)	70 (49–88)	72 (50–88)	69 (49–86)	0.0666
Sex	Male Female	43 37	25 22	18 15	1.00
CEA (ng/mL)	>3.4 ≤3.4	51 29	31 16	20 13	0.644
CA19–9 (U/mL)	>37 ≤37	55 25	28 19	27 6	**0.0497**
Localization, n (%)	Head of the pancreas Body and tail of pancreas	40 40	11 13	29 27	0.808
Histological grade	Low (wel-mod) High (por)	47 33	33 14	14 19	0.341
Tumor size (mm)	>40 ≤40	10 70	4 36	6 34	0.737
Lymphatic invasion	Present Absent	56 24	32 15	24 9	0.805
Venous invasion	Present Absent	71 9	40 7	31 2	0.294
Perineural invasion	Present Absent	61 19	35 12	26 7	0.791
EMT	EMT phenotype Non-EMT phenotype	31 49	19 28	12 21	0.817
TIL grade	High Low	38 42	24 23	14 19	0.500
Lymph node metastasis	Positive Negative	27 53	15 25	12 28	0.637
TNM stage	I-II III	57 23	34 13	23 10	0.807
Neoadjuvant chemotherapy	Yes No	6 74	3 44	3 30	0.687
Adjuvant chemotherapy	Yes No	71 9	43 4	28 5	0.477
Follow-up period (days)	Median (Range)	640 (92–3706)	469 (92–3706)	728 (140–3003)	0.103

CA19–9, carbohydrate antigen 19–9; CEA, carcinoembryonic antigen; EMT, epithelial–mesenchymal transition; mod, moderately differentiated carcinoma; por, poorly differentiated carcinoma; TIL, tumor-infiltrating lymphocyte; TNM, Tumour, Node, Metastasis; R, resectable; BR, borderline resectable; UR-LA, Unresectable - Locally Advanced; wel, well-differentiated carcinoma;.

In addition, when we examined the association between CXCR4 expression levels and other clinicopathological factors, we found that preoperative serum CA19–9 levels were significantly higher in the high CXCR4 expression group than in the low CXCR4 expression group (P = 0.0497). However, there were no statistically significant differences between the two groups in terms of age, gender, preoperative carcinoembryonic antigen levels, histology, lymphatic invasion, venous invasion, perineural space invasion, expression of EMT-related markers, TIL grade, TNM staging, and pre- and post-operative chemotherapy. Regarding preoperative treatment, only 6 patients (7.5%) received neoadjuvant chemotherapy (NAC); specifically, 3 patients each in the high CXCR4 expression group (9.1%) and low expression group (6.4%) had undergone NAC prior to surgery (P = 1.00).

### Prognostic value of CXCR4 expression in PDAC

**Fig. 2:**
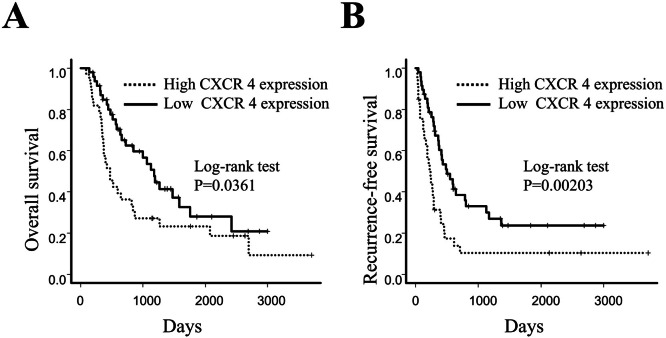
Prognostic value of CXCR4 based on Kaplan–Meier analysis. Kaplan–Meier curves for overall survival were generated for cases with high and low CXCR4 expression levels in the vasculature at sites of cancer tissue infiltration into the pancreatic surrounding fatty tissue in resected PDAC specimens. Patients with high CXCR4 expression had significantly shorter overall survival (log-rank test, P = 0.0361).

The prognostic significance of CXCR4 expression in the vasculature of primary PDAC samples was evaluated by Kaplan–Meier survival analysis and log-rank testing (Fig. 2). The median observation period was 640 days, while that for survivors was 636.5 days. The median OS was 469 days in the high CXCR4 expression group and 1176 days in the low CXCR4 expression group, with significantly shorter OS in the high expression group (log-rank test, P = 0.0361). In the survival analysis, the number of events (deaths) was 27 of 33 patients in the high CXCR4 expression group and 27 of 47 patients in the low expression group. The results of the univariate and multivariate Cox proportional hazards analyses for OS are detailed in Table 3. CXCR4 expression emerged as a significant prognostic factor (P = 0.03864). Variables that were statistically significant in the univariate analysis were subsequently incorporated into the multivariate analysis. In the multivariate model, lymphatic invasion (P = 0.0003333) and CXCR4 expression (P = 0.0342) were identified as independent prognostic indicators.

**Table 3:** Univariate and multivariate Cox proportional hazards regression analyses for overall survival.

**Factors**	**Univariate analysis**		**Multivariate analysis**	
	**Hazard ratio** **(95% CI)**	**P-value**	**Hazard ratio** **(95% CI)**	**P-value**
Age (years)	1.008 (0.98–1.036)	0.589		
Sex, n (%)	0.6255 (0.366–1.069)	0.08615		
CEA (ng/mL)	0.9287 (0.525–1.643)	0.7992		
CA19–9 (U/mL)	1.233 (0.6794–2.238)	0.4907		
Histological grade	1.414 (0.8028–2.491)	0.2302		
Lymphatic invasion	3.161 (1.759–5.677)	**0.0001178**	3.2040 (1.6960–6.052)	**0.0003333**
Venous invasion	1.294 (0.7012–2.389)	0.4094		
TIL grade	0.9822 (0.5744–1.679)	0.9476		
EMT	1.576 (0.9166–2.709)	0.09997		
Perineural invasion	1.447 (0.7278–2.877)	0.292		
TNM stage	1.901 (1.09–3.316)	**0.02355**	1.4150 (0.7832–2.556)	0.2501
Neoadjuvant chemotherapy	1.466 (0.4527–4.749)	0.5233		
Adjuvant chemotherapy	0.4391 (0.2059–0.9362)	**0.03311**	0.4604 (0.2105–1.007)	0.5203
CXCR4 expression	1.761 (1.033–3.011)	**0.03864**	1.8180 (1.0350–3.193)	**0.0342**

CA19–9, carbohydrate antigen 19–9; CEA, carcinoembryonic antigen; CI, confidence interval; EMT, epithelial mesenchymal transition; TIL, tumor-infiltrating lymphocyte; TNM, Tumour, Node, Metastasis.

### Identification of CXCR4-expressing cells in primary PDAC samples using scRNA-seq

**Fig. 3:**
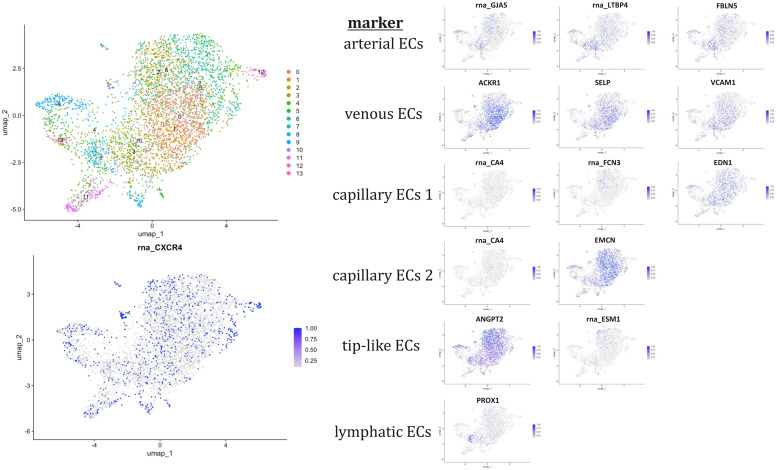
Localization of CXCR4 expression in endothelial subtypes. Single-cell RNA sequencing analysis revealed that CXCR4 expression was sparsely localized in endothelial cells (ECs) other than lymphatic ECs, specifically arterial, venous, capillary, and tip-like ECs.

scRNA-seq analysis using publicly available data revealed that CXCR4 expression was sparsely distributed among non-lymphatic endothelial cells. Specifically, CXCR4 transcripts were identified in arterial, venous, and capillary endothelial cells, as well as in tip-like endothelial cells, which play a specialized role in guiding neoangiogenesis at the vascular front (Fig. 3).

To further characterize the molecular signature of the CXCR4-expressing vasculature, we performed a DGE analysis comparing CXCR4-positive (n = 878) and CXCR4-negative (n = 3424) endothelial sub-populations. The CXCR4-positive group exhibited a distinct transcriptomic profile, with significant upregulation of 10,259 variables analyzed. It was noteworthy that beyond the marked increase in CXCR4 itself (log2 fold change >15), CXCR4-positive endothelial cells showed significant enrichment of genes involved in immunomodulation and cellular activation, including PTPRC (CD45), CD3D, FYB1, and BIN2 (Fig. S4A; all adjusted P < 0.05). Z-score-based visualizations that included violin plots and heatmaps further confirmed the robust and consistent enrichment of these markers within the CXCR4-positive sub-population across the integrated dataset (Fig. S4B, C). Specifically for PECAM1 (CD31), significant upregulation was observed in the CXCR4-positive sub-population (nominal P < 0.001, adjusted P = 0.020). Regarding VEGFA, while the adjusted P-value was 1.00 due to stringent multi-test correction, a pro-angiogenic trend was identified that exhibited a significant nominal P-value (P = 0.013) and a higher proportion of expressing cells (83.9% in CXCR4-positive cells vs. 80.5% in CXCR4-negative cells) (Fig. S5).

## Discussion

The results of this study suggest that CXCR4 is expressed primarily in the vascular endothelium in the invasive front of PDAC, and that high CXCR4 expression in the vascular endothelium is an independent prognostic factor for poor OS. Previous research primarily focused on CXCR4 expression in PDAC carcinoma cells [22,23], with no reports examining the vascular endothelium of the invasive front in PDAC. Our findings deepen the understanding of the role of CXCR4 in tumor-associated vasculature.

CXCR4 promotes vascular endothelial cell migration, proliferation, and survival through interaction with its ligand SDF-1 [22]. CXCR4 has also been reported to induce the production of angiogenic factors such as vascular endothelial growth factor by vascular endothelial cells [7]. These results suggest that high CXCR4 expression in the vascular endothelium is a robust prognostic factor for poor survival, and that increased CXCR4 expression in the tumor vascular endothelium is closely associated with tumor angiogenesis and may correlate with the progression and metastasis of PDAC. The association between microvascular CXCR4 expression and tumor aggressiveness has also been observed in other gastrointestinal cancers. For instance, Xu et al. demonstrated that CXCR4 expression in the microvasculature of hepatocellular carcinoma (HCC) is significantly correlated with vascular invasion and early recurrence [24], echoing our observations in PDAC.

Studies of surgical specimens [22] have shown that high CXCR4 expression is associated with clinicopathological features such as clinical stage and lymph node metastasis, and CXCR4 is known to play an important role in cancer cell invasion and metastasis [25]. CXCR4 expression may enhance the invasive potential of cancer cells by altering the expression of adhesion molecules and activating extracellular matrix-degrading enzymes [26]. CXCR4 has also been reported to be involved in the homing of cancer cells to specific organs [27]. These results support our finding that CXCR4 is involved in the invasive and metastatic potential of PDAC. And this finding is further supported by a meta-analysis conducted by Krieg et al. [6], which confirmed that high CXCR4 expression is a robust and consistent prognostic biomarker for poor survival in PDAC patients across various studies.

No significant differences in histological venous invasion were observed between the high and low CXCR4 expression groups. CXCR4 is known to promote angiogenesis and regulate the tumor microenvironment, but its mechanisms of action may be independent of direct vascular invasion. Therefore, the clinical impact of CXCR4 may be mediated through complex pathways rather than observable venous invasion. Furthermore, the limited sample size of this study may have obscured a subtle association between CXCR4 expression and venous invasion. To fully elucidate this complex relationship, expanded future studies are essential. In the present study, the high CXCR4 expression group exhibited significantly elevated preoperative CA19–9 levels compared to the low expression group. Since CA19–9 is a well-established surrogate marker for tumor burden and biological aggressiveness in PDAC [28], this correlation suggests that the CXCR4 pathway promotes tumor progression. CXCR4 signaling likely leads to increased tumor volume and enhanced vascular permeability, thereby facilitating the shedding of CA19–9 into the circulation.

Our scRNA-seq analysis revealed a unique expression pattern of CXCR4 within the tumor vasculature of PDAC. While a recent large-scale single-cell study across multiple cancer types identified CXCR4 as a specific marker for tip-like endothelial cells (ECs) [7], our data demonstrated a much broader distribution. In PDAC, CXCR4 expression was not limited to tip-like cells; instead, it was extensively confirmed in mature vascular endothelial cells—including arteries, veins, and capillaries—which are typically not involved in active angiogenesis (Fig. 3). Furthermore, DGE analysis revealed that CXCR4-positive endothelial cells in PDAC co-expressed such immune-related markers as PTPRC and CD3D. This molecular signature suggests that CXCR4-expressing vessels may not only facilitate angiogenesis, but also potentially function as a specialized niche for immune cell recruitment or inflammatory interactions within the tumor microenvironment (Fig. S4). To further strengthen the notion of an association between CXCR4 expression and angiogenic activity, we evaluated the correlation within our scRNA-seq dataset and witnessed that the CXCR4-positive endothelial sub-population exhibited a significantly higher expression of the canonical angiogenic marker PECAM1 (CD31) (nominal P < 0.001, adjusted P = 0.020) compared to the CXCR4-negative group. Furthermore, a pro-angiogenic trend was observed for VEGFA expression, with a significantly higher frequency of expressing cells (83.9%vs. 80.5%, nominal P = 0.013) (Fig. S5). Together, these results indicate a molecular correlation between CXCR4 signaling and the upregulation of key angiogenic factors, implicating CXCR4 as a functional marker for an active angiogenic phenotype in the PDAC microenvironment. Additionally, the upregulation of FYB1 and BIN2, both genes associated with cytoskeletal remodeling and adhesion, suggests that CXCR4 signaling is associated with functional alterations in the vasculature, such as increased motility and structural remodeling, which may be linked to the aggressive phenotype and poor prognosis observed in our clinical cohort (Fig. S4).

This pervasive expression across diverse vascular components suggests that the PDAC microenvironment induces an abnormal "re-activation" of CXCR4 signaling throughout the entire vascular network. The presence of CXCR4 in these mature vessels is potentially associated with disorganized and excessive angiogenesis, potentially facilitating rapid tumor growth and metastasis. These findings provide a robust molecular basis for the correlation between widespread CXCR4 expression and the poor prognosis observed in PDAC patients.

It must be noted that chemotherapy may modulate the tumor microenvironment and potentially affect CXCR4 expression in the residual vasculature. Previous studies have suggested that certain cytotoxic agents can induce SDF-1/CXCR4 signaling as a stress response, which might promote treatment resistance or vascular remodeling [29]. In our cohort, however, the limited number of NAC cases precluded a definitive analysis of how different regimens influenced endothelial CXCR4 levels.

Pancreatic cancer remains among the most lethal malignancies. The poor prognosis of pancreatic cancer results from its propensity for early metastasis, high invasive potential, and resistance to chemotherapy. In recent years, research on personalized therapies has been emphasized, and it has been suggested that treatment selection guided by biomarkers may improve the effectiveness of therapy [30,31]. Tumor angiogenesis plays an important role in PDAC progression and metastasis, and elucidation of the molecular mechanisms involved in angiogenesis may lead to the development of new therapeutic strategies [32]. The discovery of biomarkers is expected to aid the development of biomarker-based therapies, leading to improved prognosis; indeed, some of these therapies have already contributed to an improved prognosis [33]. The results of this study suggest the degree of CXCR4 expression in the vascular endothelium may be able to predict prognosis. Our results may also lead to the development of therapies targeting CXCR4, which we would expect to contribute to the personalized treatment of PDAC. The clinical potential of targeting this pathway is exemplified by the COMBAT trial [11], which demonstrated that the CXCR4 antagonist BL-8040, in combination with pembrolizumab, can enhance anti-tumor immune responses in metastatic PDAC.

This study has a few limitations. First, while the IHC analysis was performed using our institutional cohort, the scRNA-seq analysis relied on a publicly available database that includes diverse sample types, such as metastatic lesions, and our IHC analysis did not sub-classify vascular types (e.g., arteries or veins). In the PDAC invasive front, rigorous vascular sub-classification by IHC is technically challenging due to severe structural remodeling and intense desmoplastic reactions that obscure typical morphology. To overcome these limitations of IHC analysis, we leveraged scRNA-seq to provide a high-resolution molecular characterization, confirming CXCR4 expression across diverse endothelial subpopulations. Despite the heterogeneity between cohorts, the consistent trend of endothelial CXCR4 expression across both IHC and transcriptomic profiles provides mutual validation, supporting its reliability as a poor prognostic marker. Second, while our study identified significant prognostic trends, the relatively small sample size (n = 80) from a single-center cohort might have lacked sufficient statistical power to identify significant correlations between endothelial CXCR4 expression and TME-related factors, such as TILs and EMT. Although we did not find a significant correlation with TIL grade in this cohort, the CXCR4/CXCL12 axis is known to play a pivotal role in creating an "immune-cold" tumor microenvironment. As discussed by Sleightholm et al. [9] and Biasci et al. [10], this axis promotes the exclusion of effector T-cells and the recruitment of immunosuppressive cells, which remains a major barrier to successful therapy in PDAC. This lack of correlation in our study may be explained by the specific focus on the invasive front. Unlike the tumor center, the invasive front is a highly dynamic area characterized by active EMT and dense desmoplastic reactions, both of which may physically or chemically alter lymphocyte recruitment independently of the overall immune-cold signature of the tumor. Furthermore, our scRNA-seq analysis showed that CXCR4-positive endothelial cells co-expressed such markers as PTPRC and CD3D. This suggests that at the invasive front, CXCR4-expressing vessels may specifically interact with a subset of inflammatory cells or facilitate a specialized "inflammatory niche" rather than reflecting global TIL density. Thus, the prognostic impact of vascular CXCR4 at the invasive front might be more closely linked to its role in angiogenic progression and structural remodeling of the microenvironment instead of a general suppression of lymphocyte infiltration. To mitigate the inherent risk of overfitting in our multivariable models due to the cohort size, we strictly prioritized clinically relevant variables and verified the proportional hazards assumption. Crucially, the lack of an independent external validation cohort remains an important limitation of this study. Consequently, our findings on the prognostic value of CXCR4-positive vessels must be considered exploratory, and further large-scale, multi-center studies incorporating external validation datasets are warranted to confirm these results. Third, angiogenesis was assessed only by CXCR4 expression in endothelial cells as determined by immunohistochemical staining, not by directly measuring angiogenesis. Fourth, the number of patients who received neoadjuvant chemotherapy in this study was relatively small, which could be a relevant consideration as a NAC regimen might affect CXCR4 expression in the vasculature of residual tumors. Although NAC is increasingly becoming the standard of care, our cohort primarily reflects patients undergoing upfront surgery. Future studies on larger populations of NAC-treated patients are needed to elucidate the impact of various chemotherapy regimens on vascular CXCR4 expression and their prognostic significance in the modern treatment landscape. In addition, the findings would be strengthened and extended by investigations that directly evaluate angiogenesis. After our results have been confirmed, studies that address the development of new therapeutic strategies targeting CXCR4 will be necessary.

## Conclusion

CXCR4 expression in the vascular endothelium of the invasive front of PDAC is a poor prognostic factor. Our findings suggest that CXCR4 may be associated with PDAC progression via angiogenesis.

## Funding

This research did not receive any specific grant from funding agencies in the public, commercial, or not-for-profit sectors.

**Supplementary Fig. 1. Representative immunohistochemical staining patterns for CXCR4 scoring.** (A) Score 0: Absence of staining. (B) Score 1+: Weak, faint brown staining visible at high magnification. (C) Score 2+: Moderate brown staining where the blue hematoxylin nuclear counterstain remains visible. (D) Score 3+: Intense dark brown staining that obscures the underlying nuclear counterstain. All images were captured at × 200 magnification. Scale bar = 100 μm.

**Supplementary Fig. 2. Representative image of tumor tissue invasion sites into the pancreatic surrounding fatty tissue in resected PDAC specimen.** The areas indicated by arrows represent sites of cancer tissue invasion into the peripancreatic adipose tissue. Bar indicates 500 μm.

**Supplementary Fig. 3. Representative images of pancreatic ductal adenocarcinoma tissue.** CXCR4 expression was detected in multiple cell types at sites of cancer tissue infiltration into the pancreatic surrounding fatty tissue in resected PDAC specimens. (A, B), but mainly in the vascular endothelium (B), as confirmed by expression of CD34 (C) in the absence of D2–40 (D). A, hematoxylin and eosin staining; B, CXCR4 immunostaining; C, CD34 immunostaining; and D, D2–40 immunostaining. Bar indicates 200 μm in panel A, and 100 μm in panels B, C, and D.

**Supplementary Fig. 4. Differential gene expression analysis of CXCR4-positive and -negative endothelial cells.** (A) Volcano plot comparing gene expression profiles between CXCR4-positive (n = 878) and CXCR4-negative (n = 3424) endothelial sub-populations. The x-axis represents the log2 fold change (log_2_FC), and the y-axis represents the statistically significant adjusted P-value (-log_10_ scale). Selected genes involved in immune modulation (PTPRC and CD3D) and cellular activation (FYB1 and BIN2) are highlighted alongside CXCR4. The vertical and horizontal dashed lines indicate the thresholds for log_༒_FC >0.25 and adjusted P-value <0.05, respectively. (B) Violin plots showing the distribution of normalized expression levels of PTPRC, CD3D, FYB1, and BIN2 in CXCR4-positive versus CXCR4-negative groups. Statistical significance was determined using the Wilcoxon rank-sum test (all adjusted P-values < 0.05). (C) Heatmap of Z-score-normalized expression levels of the top differentially expressed genes across individual cells. Columns represent single cells grouped by their CXCR4 expression status. Consistent enrichment of the identified molecular signature is evident in the CXCR4-positive sub-population.

**Supplementary Fig. 5. Comparison of pro-angiogenic marker expression in CXCR4-positive and -negative endothelial cells.** Violin plots show the normalized expression levels of VEGFA and PECAM1 (CD31). Asterisks above the plots indicate statistical significance as determined by the Wilcoxon rank-sum test. For PECAM1, significant upregulation was observed in the CXCR4-positive group even after multi-test correction (nominal P < 0.001, adjusted P = 0.020). Regarding VEGFA, while the adjusted P-value was 1.00 due to the conservative Bonferroni correction, the CXCR4-positive sub-population exhibited a higher frequency of expression (83.9%vs. 80.5%), with a significant nominal P-value of 0.013. Statistical significance was determined using the Wilcoxon rank-sum test with Bonferroni correction.

## CRediT authorship contribution statement

**Shunsuke Imamura:** Writing – original draft, Visualization, Validation, Software, Resources, Project administration, Methodology, Investigation, Formal analysis, Data curation, Conceptualization. **Takeshi Uehara:** Writing – review & editing, Supervision, Project administration, Conceptualization. **Mai Iwaya:** Writing – review & editing, Resources, Investigation, Formal analysis, Data curation. **Shiho Asaka:** Writing – review & editing, Formal analysis, Data curation. **Shinsuke Sugenoya:** Writing – review & editing, Data curation. **Hiroshi Sawaguchi:** Writing – review & editing, Investigation, Formal analysis, Data curation, Conceptualization. **Yasuhiro Kuraishi:** Writing – review & editing, Data curation. **Tomoyuki Nakajima:** Writing – review & editing, Visualization, Software, Resources, Methodology, Formal analysis, Data curation, Conceptualization. **Shotaro Komamura:** Writing – review & editing, Investigation, Data curation. **Akira Nakamura:** Writing – review & editing, Data curation. **Takefumi Kimura:** Writing – review & editing, Data curation. **Yugo Iwaya:** Writing – review & editing, Data curation. **Akira Shimizu:** Writing – review & editing, Data curation. **Yuji Soejima:** Writing – review & editing, Data curation. **Hiroyoshi Ota:** Writing – review & editing, Data curation. **Tadanobu Nagaya:** Writing – review & editing, Data curation.

## Declaration of competing interest

The authors have no competing interests to declare.

## Glossary

CXCR4 (C-X-C Chemokine Receptor Type 4): A cell surface receptor that, with its ligand SDF-1/CXCL12, is involved in various biological processes including cell migration, proliferation, and angiogenesis. Its expression in tumor vascular endothelium is a focus of this study.
EMT (Epithelial-Mesenchymal Transition): A biological process where epithelial cells lose their cell polarity and cell-cell adhesion, and gain migratory and invasive properties to become mesenchymal stem cells. This process is often involved in cancer progression.
HPF (High-Power Field): A term used in microscopy to refer to the field of view when using a high-magnification objective lens.
scRNA-seq (Single-Cell RNA Sequencing): A high-throughput technology that provides transcriptomic information at the single-cell level, allowing for the analysis of gene expression in individual cells within a heterogeneous population.
SDF-1 (Stromal Cell-Derived Factor 1) / CXCL12 (CXC Motif Chemokine 12): The primary ligand for CXCR4, involved in cell migration, survival, and angiogenesis.
TMA (Tissue Microarray): A paraffin block in which up to 1,000 individual tissue cores are assembled in a grid-like pattern, allowing for high-throughput analysis of multiple tissue samples simultaneously.
TIL (Tumor-Infiltrating Lymphocyte): Immune cells (lymphocytes) that have migrated into a tumor, often indicative of the body's immune response to cancer.
Tip-like endothelial cell: Epithelial cells exhibiting characteristics of pioneering cells found at the leading edge of developing or regenerating structures. They display enhanced migratory and invasive properties, guiding processes like tissue growth or repair.
UMAP (Uniform Manifold Approximation and Projection): A dimensionality reduction technique often used for visualizing high-dimensional data, such as single-cell RNA sequencing data.

## Appendix Supplementary materials

Image, image 1 dummy alt text

Image, image 2 dummy alt text

Image, image 3 dummy alt text

Image, image 4 dummy alt text

Image, image 5 dummy alt text

Image, application 6
