# Supplementary figures and images for: Immunohistochemical analysis and scRNA-Seq identifies vascular endothelial CXCR4 expression as a predictor of poor prognosis in pancreatic ductal adenocarcinoma
Source: Transl Oncol. 2026 Jul 14;71:102916. doi: 10.1016/j.tranon.2026.102916 (PMC13382128; doi:10.1016/j.tranon.2026.102916)

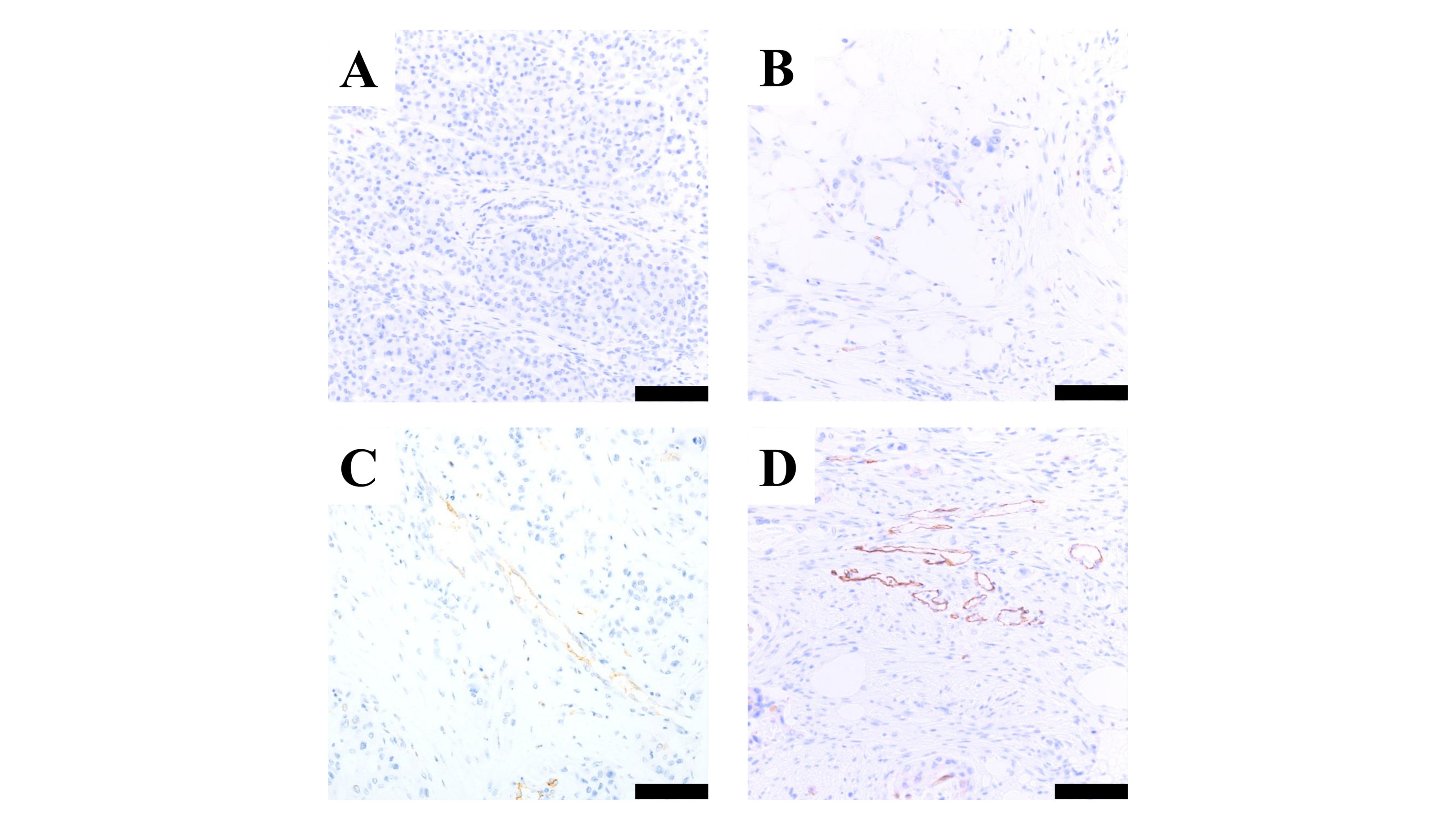

Supplement: Supplementary file 1 [file mmc1.jpg]

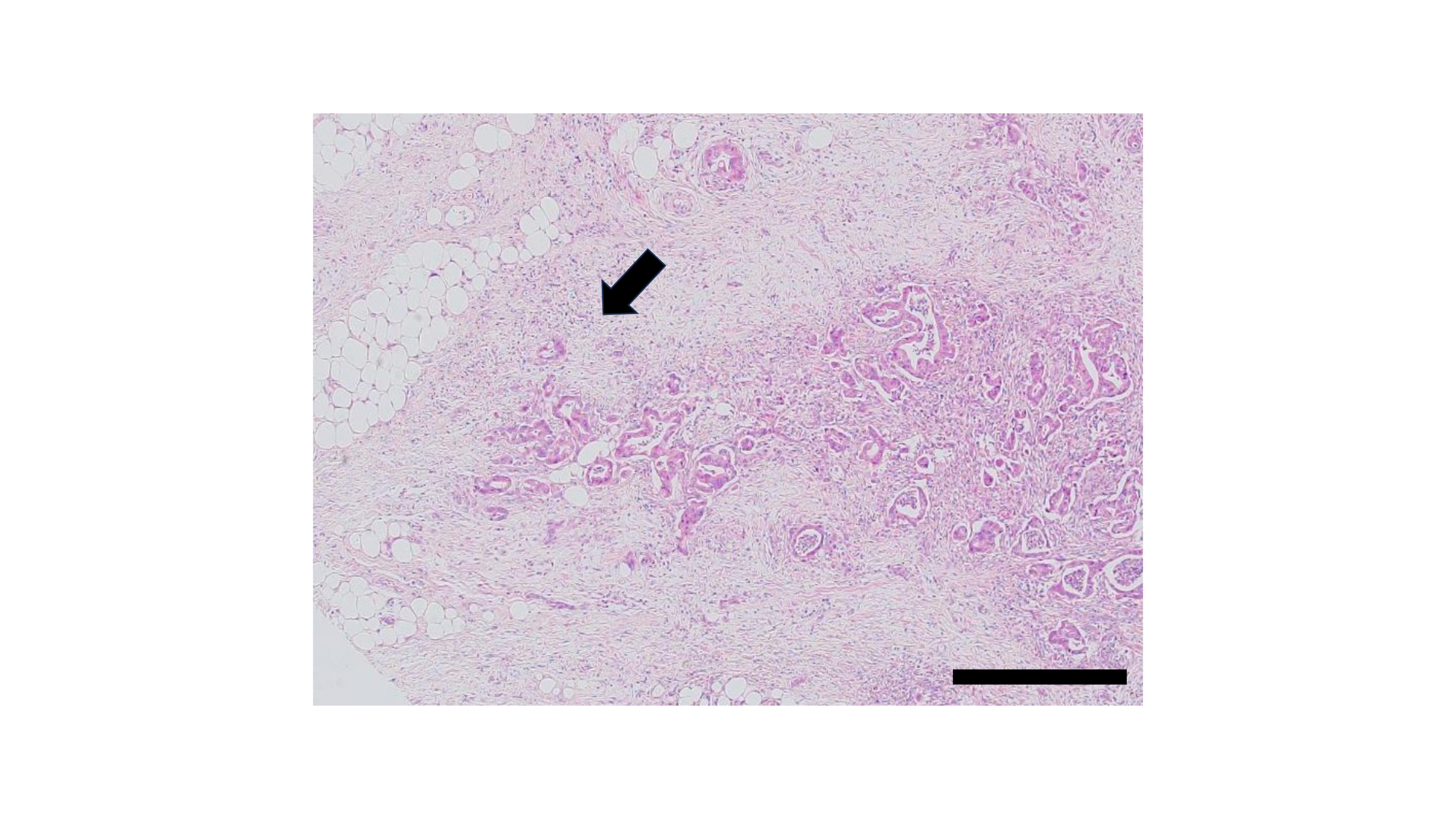

Supplement: Supplementary file 2 [file mmc2.jpg]

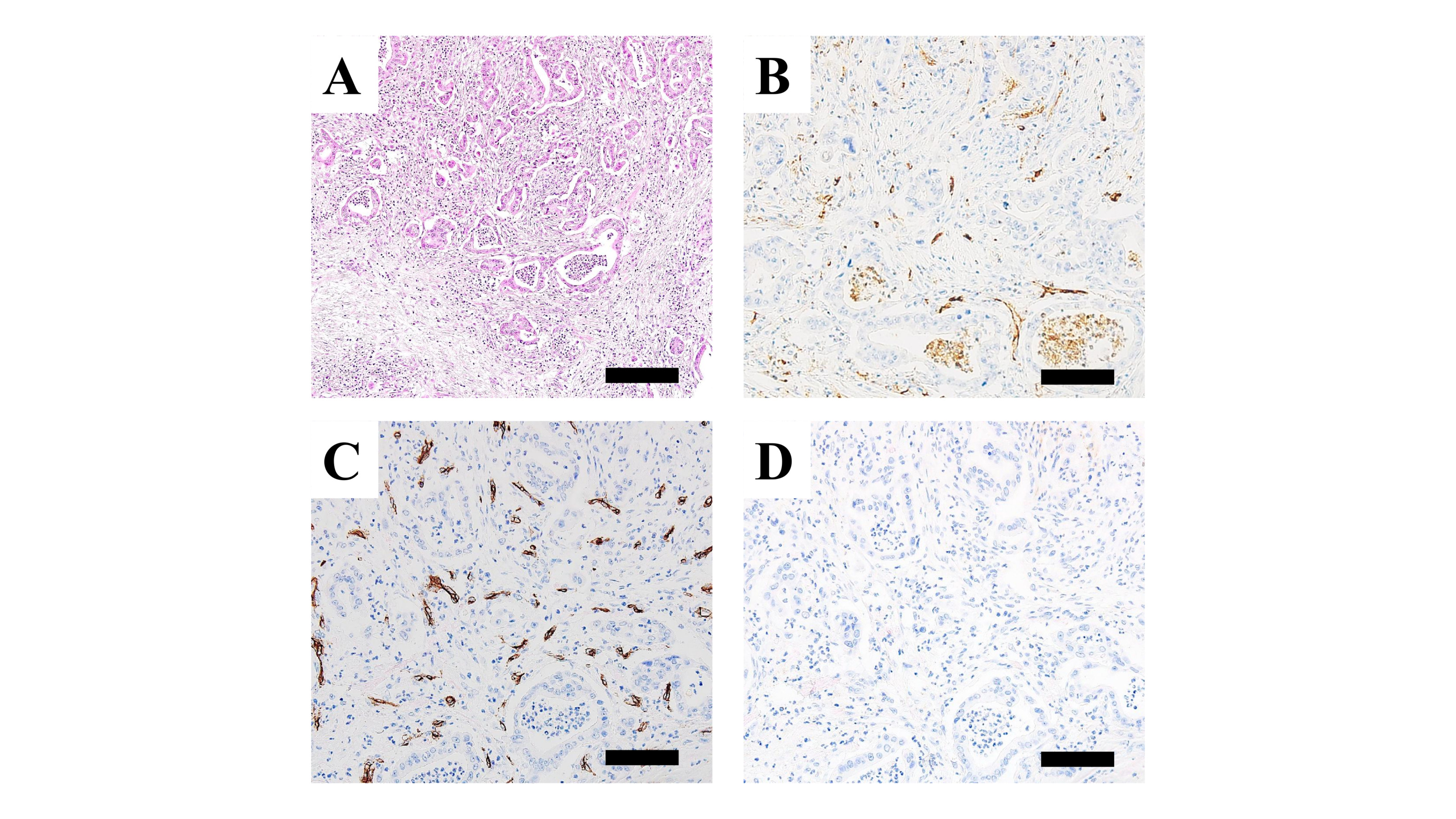

Supplement: Supplementary file 3 [file mmc3.jpg]

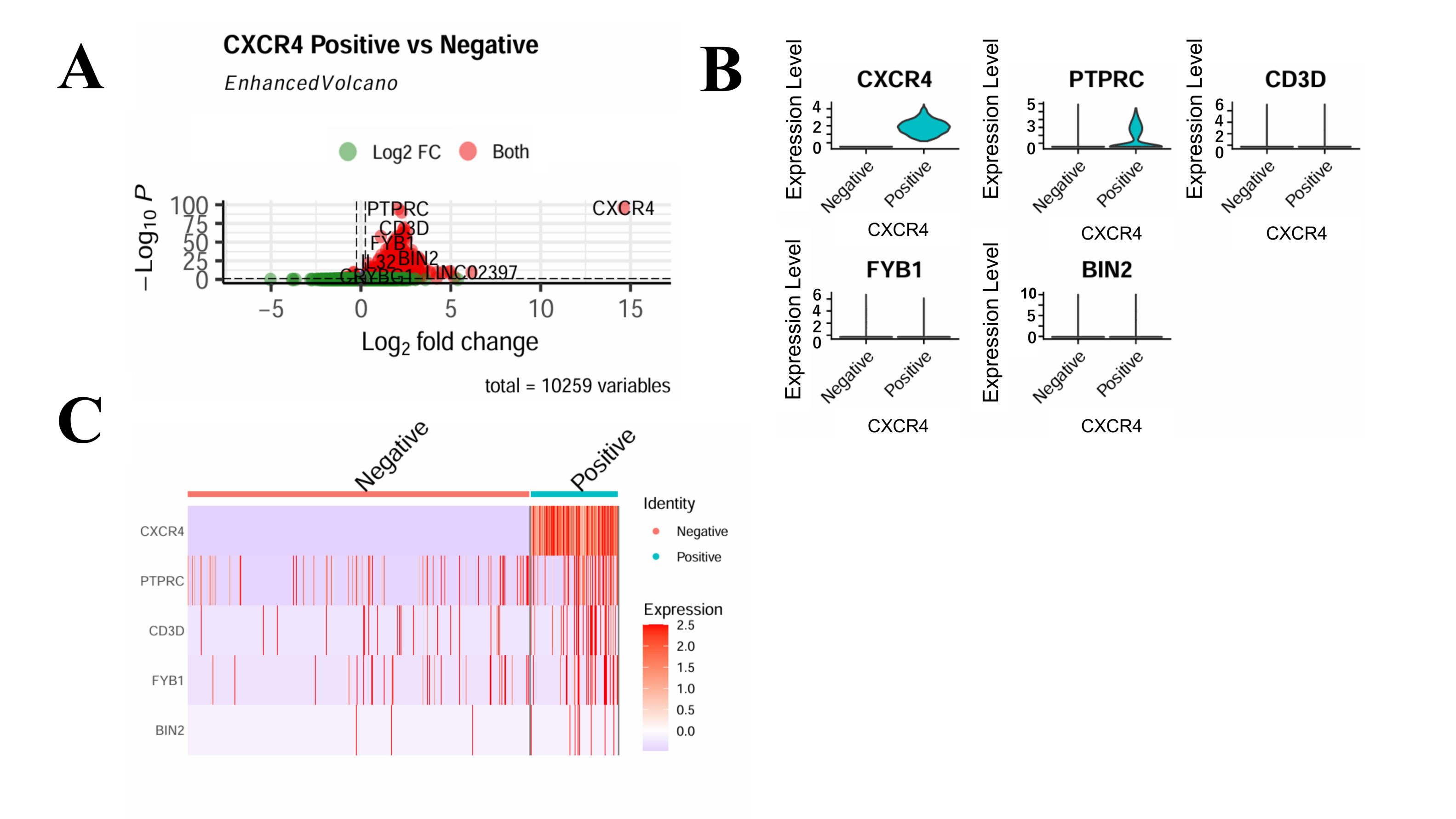

Supplement: Supplementary file 4 [file mmc4.jpg]

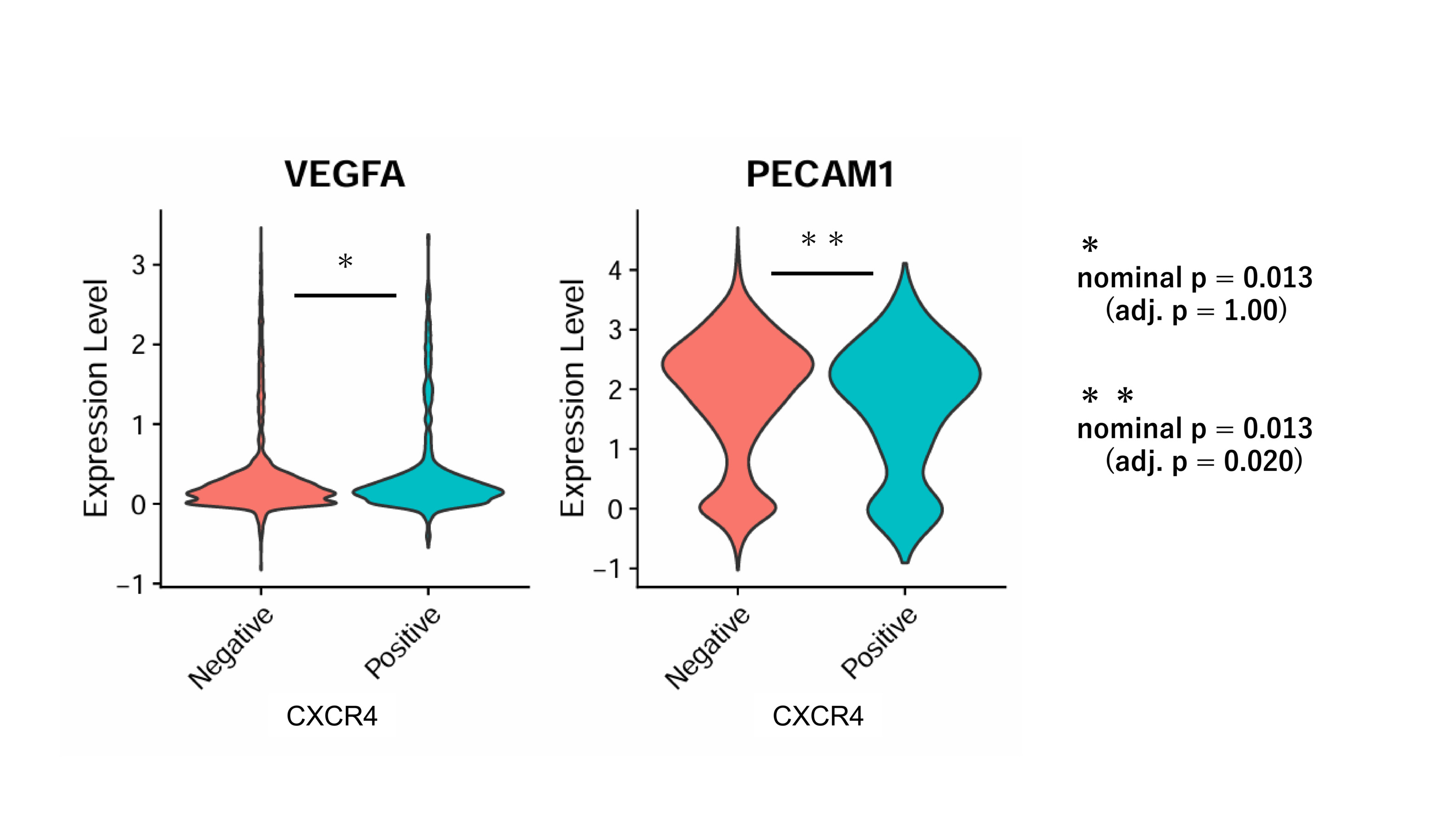

Supplement: Supplementary file 5 [file mmc5.jpg]
