# Supplementary material for: Immunohistochemical analysis and scRNA-Seq identifies vascular endothelial CXCR4 expression as a predictor of poor prognosis in pancreatic ductal adenocarcinoma
Source: Transl Oncol. 2026 Jul 14;71:102916. doi: 10.1016/j.tranon.2026.102916 (PMC13382128; doi:10.1016/j.tranon.2026.102916)
Supplement: Supplementary file 6 [file mmc6.docx]

**Supplementary Table 1** Summary of scRNA-seq dataset (GSE205013) endothelial cell analysis

|  | **Value / Count** |
| --- | --- |
| Total number of integrated batches | 17 |
| Total number of endothelial cells analyzed | 4302 |
| CXCR4-positive endothelial cells | 878 |
| CXCR4-negative endothelial cells | 3424 |
